# Polycationic Glycosides

**DOI:** 10.3390/molecules16021508

**Published:** 2011-02-11

**Authors:** Robert Engel, Ishrat Ghani, Diego Montenegro, Marie Thomas, Barbara Klaritch-Vrana, Alejandra Castaño, Laura Friedman, Jay Leb, Leah Rothman, Heidi Lee, Craig Capodiferro, Daniel Ambinder, Eva Cere, Christopher Awad, Faiza Sheikh, JaimeLee Rizzo, Lisa-Marie Nisbett, Erika Testani, Karin Melkonian

**Affiliations:** 1 Department of Chemistry and Biochemistry, Queens College of the City University of New York, 65-30 Kissena Boulevard, Flushing, NY 11367, USA; 2 Department of Chemistry and Physical Sciences, Pace University, 1 Pace Plaza, New York, NY 10038, USA; E-Mail: jrizzo@pace.edu (J.L.R.); 3 Department of Biology, Long Island University, C.W. Post Campus, 720 Northern Boulevard, Greenville, NY 11548, USA; E-Mail: kmelkoni@liu.edu (K.M.)

**Keywords:** polycationic lipid, glycoside, cyclodextrin, starch, 1,4-diazabicyclo[2.2.2]-octane, antibacterial

## Abstract

Cationic lipids have long been known to serve as antibacterial and antifungal agents. Prior efforts with attachment of cationic lipids to carbohydrate-based surfaces have suggested the possibility that carbohydrate-attached cationic lipids might serve as antibacterial and antifungal pharmaceutical agents. Toward the understanding of this possibility, we have synthesized several series of cationic lipids attached to a variety of glycosides with the intent of generating antimicrobial agents that would meet the requirement for serving as a pharmaceutical agent, specifically that the agent be effective at a very low concentration as well as being biodegradable within the organism being treated. The initial results of our approach to this goal are presented.

## 1. Introduction

Prior efforts of this laboratory have been concerned with the preparation of surfaces that serve as antibacterial and antifungal barriers for the prevention of disease transmission [1,2,3,4,5,6]. Of particular note has been the modification of carbohydrate-based surfaces (cotton, wood, paper) for the attachment of cationic lipid units that would serve as permanent disruptors of microbial cell walls as they would impinge on the modified surface [1,4]. In this manner surfaces that are permanently prophylactic regarding the transmission of disease causing microbes have been generated, based on simple carbohydrate chemistry.

While highly effective surfaces that are *prophylactic* toward disease transmission may be so constructed, such *macro*-surfaces are not applicable for the *treatment* of disease in infected patients. For such pharmaceutical applications, based on the concept of cationic lipid disruption of microbial cell walls, several structural factors need to be incorporated into the agent. First, the pharmaceutical agent must be capable of serving as a *micro*-surface bearing several cationic lipid sites on each defined molecule in order to provide a sufficient localized attack on the microbial cell wall. Further, the pharmaceutical agent must be structurally susceptible either to relatively rapid removal of itself from the treated organism after serving as an antimicrobial agent, or it must be structurally susceptible to relatively rapid degradation generating inactive species that themselves are either removed from the treated organism without deleterious effect or, alternatively, those degradation products may be usable in the ordinary metabolism of the treated organism.

Glycosides of varying sorts can be viewed as meeting these requirements for service as scaffolding for the antimicrobially active component of the potential pharmaceutical, the cationic lipid. Several types of glycosides are used in the current effort as model systems for such agents, and as potential pharmaceutical agents themselves. With regard to model systems, glycosidic derivatives of simple monosaccharides to which polycationic lipid components have been attached have been synthesized and investigated. In addition, the disaccharide sucrose has been used to serve as a scaffolding for the cationic lipid components, bearing three primary hydroxyl sites at which attachment can be accomplished within a single molecule.

More complex systems which in themselves constitute *micro*-surfaces provide the capability for cell wall destruction by incorporating several polycationic lipid components within a relatively small region of space. The difficulty with simple cationic lipids serving as antimicrobial agents stems from the unfavorable entropy factor involved, the requirement that a relatively large number of such units be organized for interaction with the cell wall. The incorporation of numerous polycationic lipid sites within a single molecular unit overcomes this unfavorable entropy factor by providing the capability for more intense local disruption of the microbial cell wall. Both linear (soluble starch) and cyclic (cyclodextrin) polyglycosides bearing numerous polycationic lipid components have been prepared and investigated in this effort. The cyclodextrin derivatives bear either six or seven polycationic lipid components with each molecule, providing a highly localized effect of a bacterial cell wall with which it impinges. In the instance of soluble starch modified in an analogous manner, a ribbon of significant length bearing the polycationic lipid units, capable of interacting with a bacterial surface over a large region.

Our goal in the present effort has been to determine the capability of glycosides modified by the attachment of cationic lipid components to serve as antibacterial agents that would be subject to chemical degradation *in vivo* in a relatively short period of time. With a practical view for the generation of such materials, it need be kept in mind that such agents would need to be prepared in significant quantity and with relatively low cost. Thus, the most readily available carbohydrate components are to be considered as the most highly desirable scaffoldings for attachment of the cationic lipid adjuncts. Efforts have thereby concentrated on the use of glucose derivatives (both monosaccharide glycosides, cyclic oligomers, and polymers), including the disaccharide sucrose.

## 2. Results and Discussion

Polycationic lipid derivatives of simple monosaccharide glycosides have been prepared from the simple glycoside in a two-step process. In the initial step, the available primary hydroxyl group(s) of the monosaccharide glycoside have been activated for a displacement reaction by selective tosylation using procedures we have previously described [1]. While the standard laboratory procedure for such tosylation generally uses pyridine as the solvent, our efforts demonstrate that an aqueous sodium bicarbonate medium also serves well, particularly for the large-scale preparations.

**Scheme 1:**
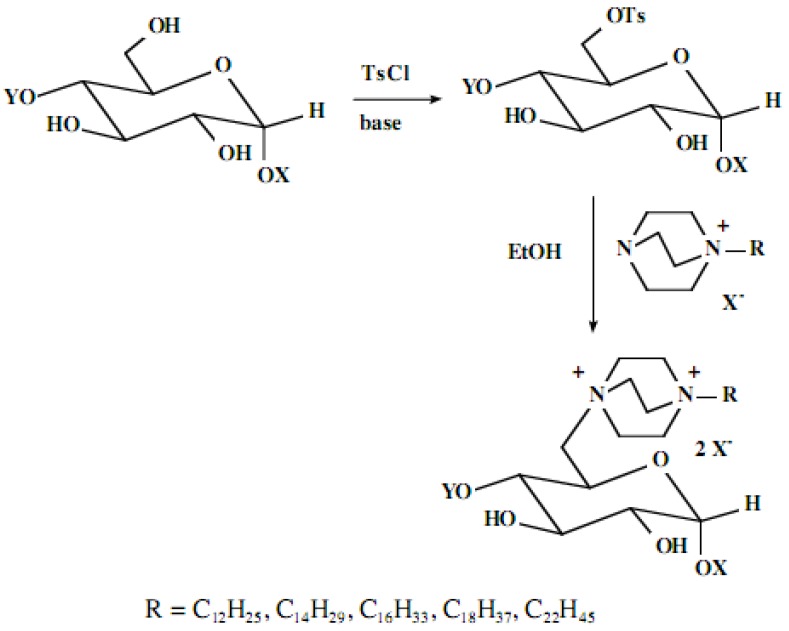
Reaction for preparation of polycationic glycosides.

In the second step of the process, displacement of the primary tosylate functionality is accomplished by treatment with a monoalkylated derivative of 1,4-diazabicyclo[2.2.2]octane (DABCO). Such derivatives of DABCO have been generated in our laboratory with selective alkylation being accomplished through the judicious choice of solvent media [7,8,9,10]. Of particular interest in the present efforts have been those bearing alkyl chains of sixteen and twelve carbon atoms, *i.e.* 1-hexadecyl-1-azonia-4-azabicyclo[2.2.2]octane halide and the twelve-carbon analogue, 1-dodecyl-1-azonia-4-azabicyclo[2.2.2]octane halide, although other chain lengths have also been of specialized interest. This two-step process is illustrated below in Scheme 1.

Our interest in these model monosaccharide glycosides has focused particularly on D-glucose modified by the attachment of monoalkylated DABCO species (in this report we focus on the use of monoalkylated DABCO units; other types of amines used for attachment to carbohydrate glycoside scaffolding will be described elsewhere). The structures of the products and the precursor monoalkylated DABCO materials are provided in Table 1, along with the yields in the particular preparations. Structural verification data (^1^H- and ^13^C-NMR spectra and quantitative elemental analyses) for these materials are provided in Table 2. Toward the construction of molecular antimicrobial *micro*-surfaces, of those discrete molecules bearing several cationic lipid components each, particular focus has been given to the cyclodextrins. 

**Table 1:** Structure of polycationic glycosides and their amine precursors.

Product Number	Product Structure	Precursor Tertiary Amine	Yield (%)
1	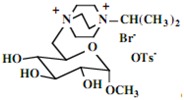	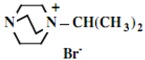	94
2	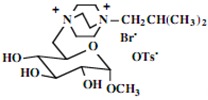	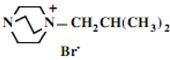	95
3	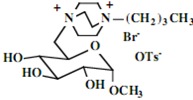	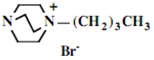	98
4	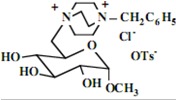	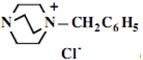	99
5	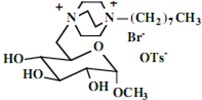	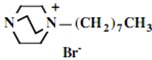	99
6	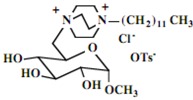	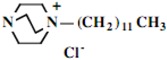	99
7	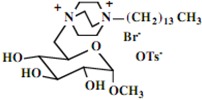	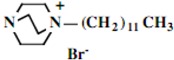	99
8	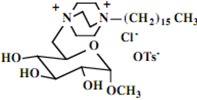	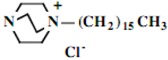	91
9	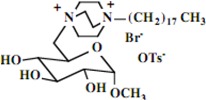	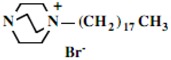	99
10	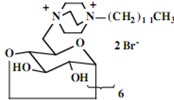	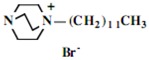	33
11	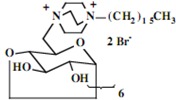	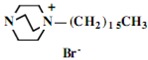	29
12	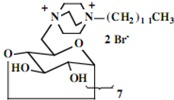	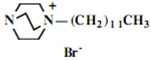	33
13	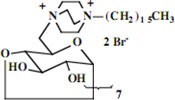	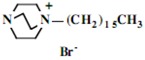	31
14	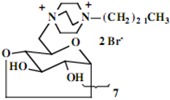	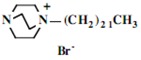	30
15	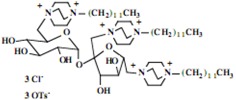	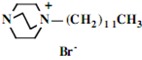	42
16	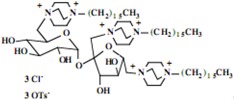	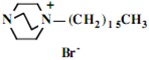	56

**Table 2:** Structural verification data for cationic glycosides.

Product Number	^1^H-NMR (D_2_O)(δ)	^13^C-NMR (δ)	Elemental Analysis
1	1.36 (6H) d, 2.31 (3H) s, 3.28-3.80 (20H) br, 7.28-7.62 (4H) *AA'BB'*	15.5, 20.5, 44.0, 47.8, 55.1, 60.6, 68.3, 69.6, 71.2, 71.6, 73.1, 99.3, 125.4, 129.5, 139.5, 142.5	Calcd. for C_23_H_39_BrN_2_O_8_S^.^8.5H_2_O: C, 37.50; H, 7.66. Found: C 37.53; H, 7.37
2	1.01 (6H) d, 2.21 (1H) br, 2.30 (3H) s, 3.35 (3H) s, 3.33-3.87 (21H) m, 7.27-7.60 (4H) *AA'BB'*	20.6, 21.9, 22.9, 44.0, 51.2, 55.1, 60.6, 69.6, 71.3, 71.6, 73.1, 74.2, 99.3, 125.4, 129.6, 139.6, 142.5	Calcd. for C_24_H_41_BrN_2_O_8_S^.^9H_2_O: C, 37.94; H, 7.83. Found: C 8.12; H, 7.79
3	0.83 (3H) t, 1.28 (2H) m, 1.64 (2H) m, 2.29 (3H) s, 3.29-3.50 (5H) br, 3.69-3.80 (16H) br, 3.81 (3H) br, 7.26-7.58 (4H) *AA'BB'*	12.7, 18.9, 20.5, 43.9, 46.2, 50.7, 55.0, 60.5, 65.1, 69.5, 71.2, 71.5, 73.0, 99.2, 125.3, 129.5, 139.4, 142.5	Calcd. for C_24_H_41_BrN_2_O_8_S^.^5H_2_O: C, 41.92; H, 7.48. Found: C 41.65; H, 7.76
4	2.34 (3H) s, 3.36 (3H) s, 3.32-3.84 (19H) br, 4.66 (2H) br, 7.32-7.64 (9H) br	20.5, 44.1, 50.6, 55.0, 60.6, 69.0, 69.6, 71.3, 71.6, 73.1, 99.3, 125.4, 126.3, 129.5, 129.6, 131.4, 133.0, 139.5, 145.5	Calcd. for C_27_H_39_ClN_2_O_8_S^.^4H_2_O: C, 50.54; H, 6.99. Found: C 50.51; H, 7.00
5	0.76 (3H) t, 1.17-1.24 (10H) br, 1.69 (2H) br, 2.29 (3H) s, 3.36 (3H) s, 3.30-3.78 (21H) br, 7.26-7.58 (4H) *AA'BB'*	13.3, 20.4, 21.3, 21.9, 25.2, 28.0, 28.1, 30.9, 43.9, 50.7, 55.0, 58.1, 60.5, 65.3, 71.2, 71.5, 73.0, 99.2, 125.3, 129.4, 139.4, 142.5	Calcd. for C_28_H_49_BrN_2_O_8_S^.^2H_2_O: C, 48.76; H, 7.74. Found: C 48.77; H, 7.80
6	0.84 (3H) t, 0.86-1.01 (18H) br, 1.24 (2H) br, 2.19 (3H) s, 3.32 (3H) br, 3.43-3.76 (21H) br, 7.08-7.57 (4H) *AA'BB'*	13.7, 20.8, 21.2, 22.5, 23.8, 28.2, 29.3, 29.4, 29.6, 29.7, 30.2, 31.8, 43.9, 46.9, 50.9, 55.0, 60.5, 69.5, 71.2, 71.2, 73.0, 99.2, 125.6, 129.1, 139.8, 141.2	Calcd. for C_32_H_57_BrN_2_O_8_S^.^2H_2_O: C, 51.53; H, 8.24. Found: C 51.46; H, 8.31
7	0.84 (3H) t, 0.86-1.01 (22H) br, 1.24 (2H) br, 2.19 (3H) s, 3.32 (3H) s, 3.43-3.76 (21H) br, 7.04-7.58 (4H) *AA'BB'*	13.9, 21.0, 21.7, 21.9, 22.8, 26.0, 26.3, 29.1, 29.7, 29.8, 30.0, 30.1, 32.2, 44.1, 46.4, 50.8, 55.1, 60.6, 64.7, 69.6, 71.3, 71.6, 73.2, 99.3, 125.8, 129.1, 139.8, 142.1	Calcd. for C_34_H_61_BrN_2_O_8_S^.^2H_2_O: C, 52.77; H, 8.47. Found: C 52.83; H, 8.52
8	0.81 (3H) t, 1.19 (26H) br, 1.69 (2H) br, 2.15 (3H) s, 3.32 (3H) s, 3.44-3.87 (21H) br, 6.94-7.55 (4H) *AA'BB'*	14.1, 21.2, 22.7, 24.5, 26.1, 26.9, 27.1, 28.9, 29.2, 29.5, 29.66, 29.68, 29.73, 29.8, 29.9, 32.7, 44.2, 45.2, 50.6, 55.3, 60.8, 69.7, 71.6, 71.7, 73.4, 99.3, 125.8, 129.1, 140.1, 141.9	Calcd. for C_36_H_65_ClN_2_O_8_S^.^5H_2_O: C, 46.56; H, 9.55. Found: C 46.53; H, 9.18
9	0.86 (3H) t, 1.24 (30H) br, 1.69 (2H) br, 2.29 (3H) s, 3.04 (2H) br, 3.31 (3H) s, 3.19-3.85 (19H) br, 7.11-7.47 (4H) AA'BB'	13.9, 21.2, 22.0, 22.8, 24.3, 26.2, 26.9, 28.9, 29.44, 29.47, 29.50, 29.6, 29.67, 29.70, 29.75, 29.8, 29.9, 32.7, 44.6, 45.7, 50.9, 55.4, 61.0, 69.9, 71.6, 71.8, 73.7, 99.6, 125.4, 128.0, 141.0, 142.1	Calcd. for C_38_H_69_BrN_2_O_8_S5H_2_O: C, 51.63; H, 9.01. Found: C 51.55; H, 9.23
11	0.86 (18H) t, 1.26 (156H) br, 1.64 (12H) br, 3.35 (12H) br, 3.55-3.90 (108H) br, 5.03 (6H) d	15.33, 23.41, 24.28, 24.31, 24.35, 27.48, 28.22, 28.71, 30.45, 30.68, 30.99, 31.41, 31.87, 31.93, 33.80, 45.99, 52.88, 60.97, 66.03, 72.19, 73.91, 75.76, 83.04, 103.94	Calcd. for C_168_H_324_Br_12_N_12_O_24_4H_2_O: C, 51.38; H, 8.52. Found: C 51.29; H, 8.63
12	0.85 (21H) t, 1.16 (126H) br, 1.60 (14H) br, 3.25 (14H) br, 3.48-3.78 (126H) br, 4.96 (7H) d	13.74, 21.02, 21.72, 22.34, 25.82, 28.64, 28.76, 28.87, 28.92, 29.04, 31.48, 44.20, 51.20, 51.86, 60.02, 65.25, 72.17, 73.61, 81.26, 102.38	Calcd. for C_168_H_322_Br_14_N_14_O_28_3H_2_O: C, 48.51; H, 7.95. Found: C 48.36; H, 8.18
13	0.86 (21H) t, 1.27 (182H) br, 1.65 (14H) br, 3.36 (14H) br, 3.45-3.81 (126H) br, 4.99 (7H) d	15.37, 23.42, 24.22, 24.35, 24.39, 27.44, 28.20, 28.75, 30.41, 30.57, 30.97, 31.28, 31.91, 31.94, 33.79, 46.01, 52.85, 60.86, 66.11, 72.23, 73.87, 75.82, 83.10, 103.96	Calcd. for C_196_H_378_Br_14_N_14_O_28_3H_2_O: C, 51.72; H, 8.50. Found: C 51.66; H, 8.58
14	0.86 (21H) t, 1.28 (266H) br, 1.66 (14H) br, 3.37 (14H) br, 3.44-3.80 (126H) br, 4.99 (7H) d	15.34, 23.41, 24.29, 24.37, 24.42, 24.58, 25.36, 27.50, 28.19, 28.83, 29.24, 30.43, 30.54, 30.61, 30.95, 31.30, 31.67, 31.93, 31.95, 33.72, 33.81, 46.03, 52.91, 60.92, 66.18, 72.27, 73. 95, 75.80, 83.17, 103.91	Calcd. for C_238_H_462_Br_14_N_14_O_28_3H_2_O: C, 55.60; H, 9.18. Found: C 55.53; H, 9.22
15	0.79-0.85 (9H) br, 1.23 (54H) br, 1.71 (6H) br, 2.14 (9H) s, 3.40-3.88 (56H) br, 6.93-7.53 (12H) AA'BB'	13.71, 14.22, 14.76, 19.73, 20.28, 20.35, 22.57, 22.86, 23.31, 23.79, 24.00, 24.43, 24.78, 25.25, 25.63, 26.04, 26.41, 26.80, 27.07, 27.65, 27.97, 28.36, 28.72, 29.23, 29.79, 30.30, 30.56, 31.18, 31.66, 32.18, 32.67, 32.94, 33.34, 44.89, 42.62, 43.01, 43.17, 44.55, 45.31, 45.87, 46.18, 46.32, 47.72, 47.93, 48.29, 49.50, 52.22, 59.48, 60.31, 65.64, 71.75, 73.47, 83.12, 99.93, 103.38, 125.56, 128.84, 141.10, 141.96	Calcd. for C_87_H_151_Cl_3_N_6_O_17_S_3_H_2_O: C, 58.91; H, 8.69. Found: C 58.78; H, 8.74
16	0.80-0.85 (9H) br, 1.21 (78H) br, 1.70 (6H) br, 2.15 (9H) s, 3.42-3.91 (56H) br, 6.95-7.55 (12H) AA'BB'	13.80, 14.13, 14.62, 19.98, 20.11, 20.31, 20.87, 22.43, 22.75, 23.29, 23.44, 23.57, 23.82, 24.10, 24.22, 24.56, 24.90, 25.23, 25.57, 25.87, 26.02, 26.39, 26.75, 27.12, 27.27, 27.54, 27.80, 28.04, 28.23, 28.58, 28.65, 29.18, 29.70, 30.44, 30.67, 31.32, 31.65, 31.99, 32.27, 32.31, 32.54, 32.86, 33.29, 44.65, 42.71, 43.09, 43.18, 44.67, 45.27, 45.73, 46.03, 46.38, 47.52, 47.99, 48.18, 49.82, 52.34, 59.67, 60.21, 65.48, 71.47, 73.76, 82.66, 99.71, 102.84, 125.73, 129.29, 140.02, 141.80	Calcd. for C_99_H_175_Cl_3_N_6_O_17_S_3_H_2_O: C, 61.23; H, 9.19. Found: C 61.17; H, 9.28

Several intriguing points are to be noted in these syntheses. First, the isolated products, being polycationic organic salts, as with the previously noted polycationic organic salts we have investigated [6,7,8,9,10] are significantly hydroscopic. In all analyses, account must be taken for the formation of solid materials in which varying amounts of water are agglomerated. 

Further, the yields for the syntheses of the model monosaccharide polycationic glycosides are excellent, a result not unexpected with simple nucleophilic substitution reactions of this type, and in keeping with our prior observations. However, the yields of isolated polycationic cyclodextrin derivatives are rather low, and *the isolated salts in the direct syntheses bear only halide ion and no tosylate ion.* The absence of tosylate ion was an intriguing observation from the NMR spectra of the precipitated salts. Further investigation of the solutions from which these salts were isolated in yields of approximately 30% indicated that the tosylate and tosylate/bromide ion conjugates remain soluble; the salts precipitating readily under our conditions are those units bearing only bromide ion (we have also investigated the ion exchange of these materials to generate salts with a single type of anion, but this does not affect the ultimate results.) Apparently, under the solvent conditions used, the pure bromide salts precipitate readily while the salts bearing one or more tosylate ions, possibly held within the cyclodextrin cavity, remain in solution. We have proceeded in the investigations of the antimicrobial characteristics using the bromide salts.

Antibacterial and antifungal activity of the synthesized salts were measured using the procedure described in the *Experimental* section. As a general reference for antibacterial activity, the effect against *S. aureus* was noted. As may be noted in Table 3, the MIC for each of the simple monosaccharide glycosides with *S. aureus* is relatively high, a consequence of a significant entropy factor involved with bringing an effective concentration of cationic lipid to impinge on the bacterial cell wall. However, with the modified cyclodextrins, much lower values for MIC are to be noted, a consequence of multiple impingement of cationic lipid units (six or seven) with the bacterial cell wall within a small surface area of that bacterial cell wall.

**Table 3:** Activity of synthesized materials toward *S. aureus* and other organisms (MIC; mg/mL).

Compound	*S. aureus*	*P. aeruginosa*	*A. niger*	*C. albicans*
**1**	>5.8	-	-	-
**2**	>5.9	-	-	-
**3**	>5.9	-	-	-
**4**	3.11	-	-	-
**5**	>6.5	-	-	-
**6**	>6.6	12.5	-	-
**7**	1.99 × 10^−1^	-	-	-
**8**	>7.2	-	4.9 × 10^−2^	2.4 × 10^−2^
**9**	>7.9	-	-	-
**10**	4.6 × 10^−2^	-	-	-
**11**	2.3 × 10^−2^	-	-	-
**12**	2.3 × 10^−2^	3.9 × 10^−1^	2.0 × 10^−1^	4.9 × 10^−2^
**13**	2.3 × 10^−2^	1.56	4.9 × 10^−2^	2.4 × 10^−2^
**14**	2.4 × 10^−2^	-	-	-
**15**	1.3	-	-	-
**16**	2.4	-	-	-
**modified soluble starch**	1.3 × 10^−7^	-	-	-

In addition to the Gram-positive bacterium studied, *S. aureus*, for selected systems activity against a Gram-negative bacterium, *Pseudomonas aeruginosa*, and two fungi, *Candida albicans* and *Aspergillus niger*, have been investigated. These results (MIC values) are also provided in Table 3.

The construction of a polycationic glycoside from potato starch (Aldrich, ACS Reagent starch, soluble, partially hydrolyzed) was performed in the same manner as it was used to accomplish functionalization of both the monosaccharide derived glycosides and the cyclodextrin derivatives. With a system such as soluble starch, a long chain bearing regularly spaced pendant alkyl-substituted DABCO units could be constructed that would have the capability of impinging on a bacterial cell wall with a very large number of cationic lipids. As noted in Table 3, such a system exhibits a very significantly lower MIC toward *S. aureus* (a factor of 5 × 10^4^ more efficient than the cyclodextrin derivatives) and holds the greatest potential to serve as a potential antimicrobial pharmaceutical agent. 

## 3. Experimental

### 3.1. General

All chemicals and solvents used in these syntheses and purifications were of commercial reagent quality and were used without further purification. All ^1^H- and ^13^C-NMR spectra were measured with the samples in commercial deuterated solvents (D_2_O) using a Brüker 400-MHz DPX400 instrument. Elemental analyses were performed by Schwarzkopf Microanalytical Services of Woodside, NY, USA.

### 3.2. Syntheses of cationic lipid appended glycosides

*General procedure - monosaccharide glycosides and starch.* The appropriate glycoside (approximately 5.0 g) was dissolved in water (100 mL) to which a small excess of NaHCO_3_ was added (1.1 mol amount for monosaccharide glycosides, and 1.1 molar amount based on calculation of monomeric D-glucose units of starch), followed by 1 molar amount of *p*-TsCl. The mixture was stirred vigorously for 1 h at ambient temperature to allow the formation of the appropriate 6-*O*-tosyl derivative. After this time 1 molar amount (based on the tosylation performed) of the selected monoalkylated DABCO derivative was added, and the reaction mixture was stirred under reflux for 14 h. After cooling, the solvent was evaporated under reduced pressure leaving a viscous liquid that became solid when residual solvent was removed under high vacuum. The impure solid was treated with 95% ethanol and any insoluble materials were removed by suction filtration. The remaining solvent was then evaporated under reduced pressure and the remainder was dried under high vacuum to provide the target material. Structures, yields, and analytical data for these materials are presented in Table 1 and Table 2.

*General procedure - cyclodextrins.* The appropriate cyclodextrin hydrate (0.003 mol) was added to a solution of 10% NaHCO_3_ in water (50 mL). This was followed by the addition of a solution of *p-*toluenesulfonyl chloride (0.025 mol) in 10% aqueous NaHCO_3_ solution (75 mL). The solution was allowed to stir at ambient temperature overnight after which the solution was filtered with suction and the filtrate evaporated under reduced pressure to yield the crude polytosylate as a viscous liquid. The crude polytosylate was dissolved in acetonitrile (75 mL) and a solution of the appropriate monoalkyated DABCO reagent (0.025 mol) dissolved in acetonitrile (50 mL), was added slowly with stirring. The reaction mixture was heated at reflux with stirring for 6 days after which the tan powder that had formed was collected by suction filtration, washed with portions of ethyl acetate and diethyl ether, and dried under high vacuum. Structures, yields and analytical data for these materials are presented in Table 1 and Table 2.

### 3.3. Evaluation of antimicrobial activity of salts

A measured quantity of the salt to be investigated and a stock solution of bacteria (5 µL, ~5 × 10^4^ cells, *S. aureus*) were added to Luria-Bertani (LB) broth (2 mL). (Corresponding additions were made for *P. aeruginosa* and the fungi investigated.) The growth medium was incubated overnight at 37 °C in a shaking water bath. The absorbance (abs, indicating turbidity of the medium) at 600 nm was recorded. The results were noted as percent growth:

(abs of sample/abs of blank) × 100.

The results are reported in Table 3. Minimum inhibitory concentrations (MIC) were determined by preparing serial dilutions of each salt and adding the same amount of bacteria to each dilution. dilutions were incubated overnight at 37 °C in a shaking water bath and growth was observed in comparison to a blank.

## 4. Conclusions

Cationic lipid units attached through the 6-position of monosaccharide glycosides exhibit a moderate antibacterial effect. While relatively low in activity, these species are nonetheless more active toward bacteria than the simple alkyl-substituted cationic lipids. 

The concept of grouping several cationic lipid sites within a single molecule to increase the antibacterial efficacy of the material has been borne out in this investigation. The use of cyclodextrin scaffoldings through which six or seven cationic lipids are organized to impinge on a bacterial cell wall simultaneously within a small region of surface has been found to be an effective approach to increasing antibacterial activity for the cationic lipid species. The glycosidic linkages of the cyclodextrin derivatives provides the capability for hydrolysis of the unit to individual fragments that can then be disposed of by a host organism. This structural characteristic of the cyclodextrins enables one to overcome the deleterious entropy factor involved in organizing cationic lipids for destruction of a bacterial cell wall, and provides an effective approach for the design of non-antibiotic antibacterial agents.

Further, it is of particular note that the use of soluble starch modified by the incorporation of polycationic lipid units along the entire chain length provides a significantly greater antibacterial effect even compared to the cyclodextrin derivatives. The impingement on the bacterial cell wall by such a material will provide an extended swath of disruption to that cell wall sufficient to produce cytotoxicity at a very low concentration.

As noted, the goal of the current effort was the determination of the potential of simple, readily available glycosidic materials to be modified to serve as antibacterial agents, potentially for *in vivo* applications. The presented results demonstrate this potential to be real. Simple glycosides, readily available in sizable quantity, are capable of being modified by the rapid chemical attachment of cationic lipid adjuncts to serve the desired purpose. Most significantly, the use of a simple starch as a scaffolding for the cationic lipid adjuncts provides a significantly high efficacy as an antibacterial agent that warrants continuing investigation toward serving this purpose. Our current goals are the optimization of the starch/adjunct structure for this end, along with determination of the conditions and rates for *in vitro*, and ultimately *in vivo* degradation of the agent.
